# Inhibition of the ER stress IRE1α inflammatory pathway protects against cell death in mitochondrial complex I mutant cells

**DOI:** 10.1038/s41419-018-0696-5

**Published:** 2018-05-31

**Authors:** Meghan S. Soustek, Eduardo Balsa, Joeva J. Barrow, Mark Jedrychowski, Rutger Vogel, Jan Smeitink, Steve P. Gygi, Pere Puigserver

**Affiliations:** 1 Department of Cancer Biology, Dana-Farber Cancer Institute, Harvard Medical School, Boston, MA 02215 USA; 2 Department of Cell Biology, Harvard Medical School, Boston, MA 02215 USA; 3 Radboud Center for Mitochondrial Medicine, Radboud University Medical Center, Nijmegen, 6500HB The Netherlands

## Abstract

Mitochondrial mutations cause bioenergetic defects associated with failures to use the electron transfer chain and oxidize substrates. These defects are exacerbated under energetic stress conditions and ultimately cause cell deterioration and death. However, little is known about cellular strategies that rescue mitochondrial stress failures and maintain cell survival under these conditions. Here, we have designed and performed a high-throughput chemical screen to identify small molecules that rescue human mitochondrial complex I mutations from energetic stress-induced cell death. The top positive hits were a series of sulfonylureas that efficiently maintain prolonged cell survival and growth under energetic stress conditions. The addition of galactose instead of glucose, to experimentally force mitochondrial respiration, triggered an initial ER stress response that was associated with IRE1α-dependent inflammatory signals including JNK and p38 MAP kinases in mutant cells. Sulfonylureas, similar to inhibition of IRE1α and p38 MAP kinase, potently blocked this ER stress inflammatory and cell death pathway and maintained viability and cell growth under severe energetic stress conditions. These studies reveal that sulfonylureas and specific inhibition of the IRE1α inflammatory pathway protect against cell death and can be used to rescue bioenergetic failures in mitochondrial complex I-mutated cells under stress conditions.

## Introduction

Mitochondrial diseases encompass a large group of heterogeneous disorders stemming from mutations in either nuclear or mitochondrial genomes and result in an overall impairment in the oxidative phosphorylation (OXPHOS) system^1^. It is estimated that 1:5000 people are affected by a mitochondrial disorder, and currently there are no available cures^2^. Of the different complexes that make up the mitochondrial respiratory chain, complex I (CI) is the largest and mutations in CI are the most common OXPHOS defects in patients^3^. Mutations in CI cause lowered ATP production, increased reactive oxygen species (ROS), imbalances in NAD+/NADH ratio and impaired mitochondrial membrane potential^1,4^. Currently, many treatments are aimed at rescuing OXPHOS by bypassing CI and utilizing CI-independent pathways by using compounds such as CoQ1 or cell membrane-permeable prodrugs of succinate^5,6^.

While OXPHOS is the major pathway for generating ATP, many different cell types predominantly utilize glycolysis in vitro, making it difficult to study defects in mitochondrial respiration. To circumvent this challenge, cells can be cultured in media containing galactose instead of glucose. This forces cells to use OXPHOS instead of glycolysis for ATP production^7^. While cells without mitochondrial defects transition from glycolysis to OXPHOS seamlessly, cells harboring mitochondrial mutations either fail to proliferate or undergo cell death as a consequence of impaired OXPHOS^8^. We and others have used this galactose-sensitivity assay to design high-throughput screens to identify small molecules or genes that can either redirect oxidative metabolism or boost mitochondrial function to increase cell viability^9,10^. While glucose deprivation is employed as a method to force cells to utilize OXPHOS, it has also been shown to trigger ER stress and the unfolded protein response (UPR)^11,12^. Three sensors in the ER become activated as a result, these include protein kinase R (PKR)-like ER kinase (PERK), activating transcription factor 6 (ATF6) and inositol-requiring enzyme 1 (IRE1). Depending on the duration and strength of the stimulus, these factors activate different effectors that either ameliorate stress and lead to cell survival, or initiate cell death^13^. For example, under severe or sustained ER stress, IREα1 can recruit TRAF2 and ASK1 consequently activating JNK and p38 MAPKs ultimately leading to initiation of inflammation and cell death^14,15^.

Here we identified a subset of sulfonylureas, K+ (ATP) channel inhibitors, which convey significant rescue of cybrid cells harboring a human mitochondrial CI-mutation using a positive high-throughput chemical screen. Interestingly, while sulfonylureas did not alter mitochondrial bioenergetic function, they strongly inhibited IRE1α pro-apoptotic and inflammatory signaling through p38 and JNK kinases. These studies (1) reveal that sulfonylureas protect against cell death and can be used to maintain cell survival in mitochondrial complex I-mutated cells under conditions of energetic and (2) highlight that cells harboring mitochondrial CI-defects are more susceptible to ER stress-induced inflammation and cell death.

## Results

### Sulfonylureas rescue a human mitochondrial complex I mutation from energetic stress-induced cell death

**Fig. 1:**
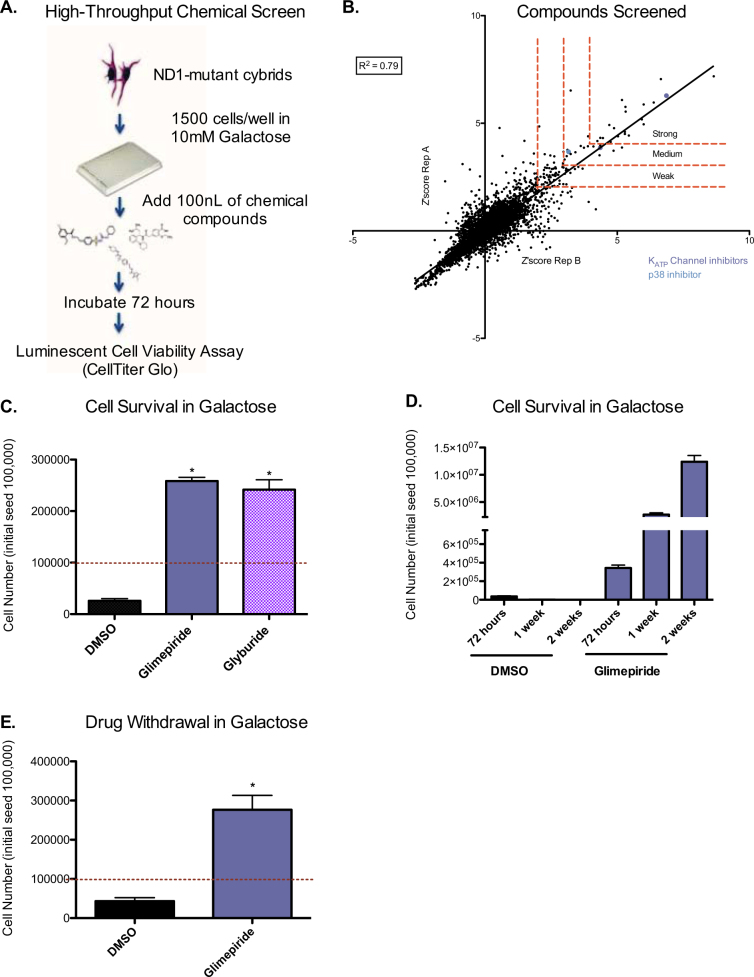
High-throughput chemical screen reveals sulfonylureas rescue complex I mutants from energetic stress-induced cell death. **a** Schematic of high-throughput chemical screen. **b** Scatterplot of *Z′* scores of all small molecular compounds tested. Cutoffs for characterizing compounds as strong, medium, or weak hits are indicated (red dashed line). Replicate A (*y*-axis) is plotted against replicate B (*x*-axis). Sulfonylureas (purple) and p38 (blue) inhibitors are identified. **c** Cell survival of ND1 cells cultured in galactose treated with either glimepiride or glyburide for 72 h. Red dashed line indicates initial seeding density. Results shown as mean ± SEM; *p < 0.05 (*n* = 3). **d** ND1 cell survival and growth over two-weeks with glimepiride. Results shown as mean ± SEM (*n* = 3). **e** Cell survival of ND1 cells having been treated for 7 days with glimepiride in galactose conditions followed by either continuous culturing in glimepiride or DMSO in galactose for an additional 72 h. Results shown as mean ± SEM; *p < 0.05 (*n* = 3)

In order to identify chemical compounds that rescue mitochondrial CI-mutant cells from energetic stress-induced cell death, we developed a high-throughput chemical screen in which human cybrid cells harboring a mutation (3796A>G) in the mitochondrial protein ND1 were seeded in galactose and cell viability was assessed 72 h later (Fig. 1a). As described previously, ND1-mutant cybrid cells die in galactose media within 72 h while control cells survive and proliferate^9^. A diverse library of 5056 compounds was screened in duplicate and a *Z′* score was calculated for each compound replicate. Based on the *Z′* score, compounds were classified as strong, medium, or weak hits. One of the top hits from the screen was the sulfonylurea glimepiride. Interestingly, an additional K+ (ATP) channel inhibitor, glyburide, was also identified as a medium hit (Fig. 1b). Both compounds retested in house to similar degrees (Fig. 1c). Furthermore, treatment with glimepiride increased cell proliferation and prolonged rescue from cell death in galactose over the course of several weeks (Fig. 1d). To investigate the effect glimepiride has on wildtype cells, we cultured control cells in galactose with glimepiride. Short-term treatment of control cells in galactose with glimepiride had no effect on cell proliferation at 72 h or after 1 week, although treatment did lead to a slight decrease in cell growth after 2 weeks of treatment (Supplementary 1). We next tested cell survival dependency on glimepiride by culturing ND1-mutant cells in galactose with drug for 1-week then either removing the drug or continuing treatment. Drug withdrawal resulted in cell death at an additional 72 h, suggesting that glimepiride is required to maintain cell viability and proliferation (Fig. 1e).

Sulfonylureas belong to a subset of antidiabetic drugs that are widely used to treat type-2 diabetes. In pancreatic beta cells, they promote insulin secretion by binding to the sulfonylurea receptor and inhibiting the K+ (ATP)-dependent channel^16^. This leads to an increase in intracellular calcium levels and sequentially insulin secretion. Reportedly, these drugs have a high specificity for the sulfonylurea receptor and are typically used in vitro in the nanomolar range;^17^ however, based on the confines of the chemical screen compounds were administered in the micromolar range. Specifically, sulfonylureas were used at 25 μM, making it unlikely at this concentration that they increase cell survival by exclusively acting on the K+ (ATP) channels. Nevertheless, in an effort to confirm this, we performed a dose-response curve to determine the working range of glimepiride in conferring ND1-mutant rescue. Cell survival increased with increasing concentrations of glimepiride, the most significant rescue occurring at the highest concentrations (25–50 μM). No rescue was observed at concentrations below 10 μM (Supplementary 2A). To further explore the involvement of the K+ (ATP) channel in cell survival, we used CRISPR/CAS9 genome editing to deplete the Kir6.2 channel that controls potassium efflux and is regulated by the sulfonylurea receptor. When 60–70% depletion was obtained at the protein level it did not result in an increase in ND1 cell survival (Supplementary 2B and C). Therefore, it is unlikely that the rescue observed is through the K+ (ATP) channel.

### Activation of p38 MAP kinase mediates energetic stress-induced cell death in ND1 cells

**Fig. 2:**
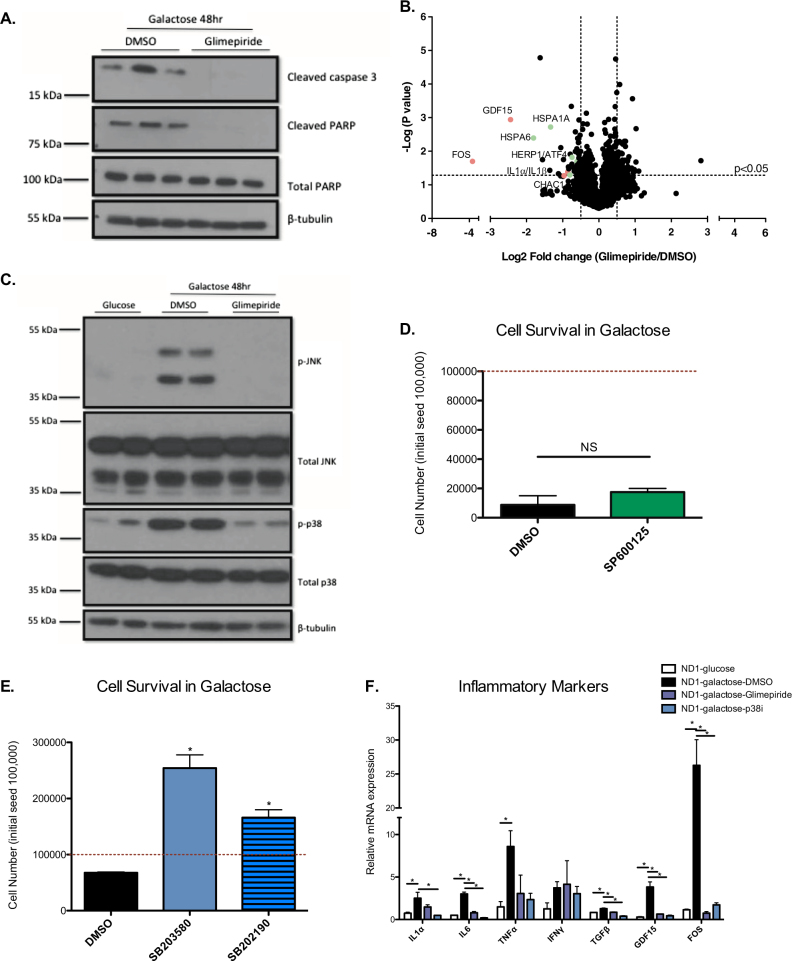
Sulfonylureas inhibit pro-apoptotic signaling triggered by galactose media. **a** Immunoblot of Cleaved Caspase 3, Cleaved PARP, and total PARP levels in ND1 cells treated with DMSO or glimepiride in galactose for 48 h. **b** Volcano plot of global proteomics analysis of cells treated with DMSO or glimepiride in galactose. Values are expressed as glimepiride/DMSO. Dashed lines indicate cutoff for *p* < 0.05 and fold change is > ± 0.5 (*n* = 3). Proteins involved in inflammatory processes are indicated in red; proteins involved in ER stress signaling are indicated in green. **c** Immunoblot of total and phosphorylated levels of MKK4, JNK, and p38 levels of ND1 cells in glucose or galactose with DMSO or glimpiride for 48 h. **d** Cell survival of ND1 cells treated with DMSO or SP600125 in galactose. Red dashed line indicates initial seeding density. Results shown as mean ± SEM (*n* = 3); NS not significant. **e** Cell survival of ND1 cells treated with DMSO, SB203580, or SB212190 in galactose. Red dashed line indicates initial seeding density. Results shown as mean ± SEM (*n* = 3); *p < 0.05. **f** mRNA levels of inflammatory markers from ND1 cells cultured in glucose or galactose with DMSO, glimepiride or SB203580 (ND1-p38i) analyzed by qPCR. Results shown as mean ± SEM (*n* = 3); *p < 0.05

Although ND1 cells die within 72 h, we sought to determine if apoptotic signaling could be detected at an earlier time point; therefore, we measured cleaved caspase-3 and cleaved PARP protein levels in ND1-mutants treated either with DMSO or glimepiride in galactose at 48 h. ND1-mutants treated with DMSO were committed to cell death at 48 h, while cells treated with glimepiride were strongly protected from proapoptotic signaling (Fig. 2a). To gain further insight into the mechanism of rescue, we performed global proteomics analyses on ND1-mutants cultured in galactose for 48 h and found that FOS, along with other inflammatory proteins such as GDF-15, IL1α, and IL1β, were significantly downregulated with glimepiride treatment (Fig. 2b). MAPKs control the metabolic fate and viability of cells by mediating apoptotic and inflammatory signaling during extracellular stresses such as energetic stress^18^. Interestingly, we identified the p38 inhibitor SB203580 as a medium hit from the chemical screen (Fig. 1b). At 48 h, both active p-JNK and p-p38 levels where highly induced in ND1 cells cultured in galactose compared to cells cultured in glucose or ND1 cells cultured in galactose with glimepiride (Fig. 2c). We found that this increase in MAPK activation was specific to ND1-mutant cells, as control cells did not induce activation of either MAPK when cultured in galactose (Supplementary 3A). To determine if JNK or p38 activation was the underlying cause of cell death, we treated ND1-mutant cells with JNK or p38 inhibitors and measured cell survival. At 72 h, we did not observe any increase in cell viability with SP600125 but did see a significant increase in cell survival in cells treated with either p38 inhibitor, SB203580 or SB202190, when compared to DMSO controls (Fig. 2d, e). We next measured mRNA levels of several inflammatory markers that have been shown to increase with p38 activation. Galactose induced several of these markers such as *IL1*α, *IL6*, *TNF*α, *TGFβ*, *GDF-15*, and *FOS*, and most of which, with the exception of *TNF*α were decreased with SB203580 and glimepiride treatment (Fig. 2f). As expected, these genes were not induced in control cells when cultured in galactose, demonstrating that the inflammatory pathway is only activated in mitochondrial CI-deficient cells (Supplementary 3B). This data suggests that p38 inhibition plays a crucial role in cell survival under energetic stress conditions, and glimepiride acts as a potent inhibitor of the p38-signaling apoptotic pathway in ND1-mutant cells.

Both p38 inhibition and treatment with glimepiride led to a significant decrease in GDF-15. Interestingly, GDF-15 has been described as a biomarker for mitochondrial dysfunction^19^. To gain further insight into how glimepiride may alleviate mitochondrial-dependent energetic stress, we investigated whether treatment with glimepiride increased ETC activity and ATP synthesis. ATP/ADP ratios showed no significant changes in ATP levels with drug treatment; additionally, basal oxygen consumption levels from isolated mitochondria were not increased (Supplementary 4A and B). We further assessed mitochondrial ETC activities of complexes I, II, and IV along with citrate synthase (CS) activity and found CI, IV, and CS levels to be unchanged while CII activity was slightly reduced (Supplementary 4C and D). Since ROS is a known activator of p38, we measured ROS levels and found that while galactose conditions increases ROS levels compared to glucose, glimepiride did not alter ROS levels (Supplementary 4E)^20,21^. Mitochondrial membrane potential (MMP) is a major determinant of cell death and decreases in MMP are indicative of mitochondrial dysfunction. Culturing cells in galactose led to a marked decrease in MMP at 48 h compared to glucose, while glimepiride treatment led to an increase in MMP; however, glimepiride was unable to fully restore MMP to the levels observed under glucose conditions (Supplementary 4F). Thus, we concluded that sulfonylureas do not increase ND1-mutant cell survival in galactose by increasing OXPHOS capacity or by reducing ROS, but likely inhibit cell death and relieve mitochondrial stress through a different cellular mechanism.

### CI missense mutant cells are vulnerable to mitochondrial energetic-dependent ER stress

**Fig. 3:**
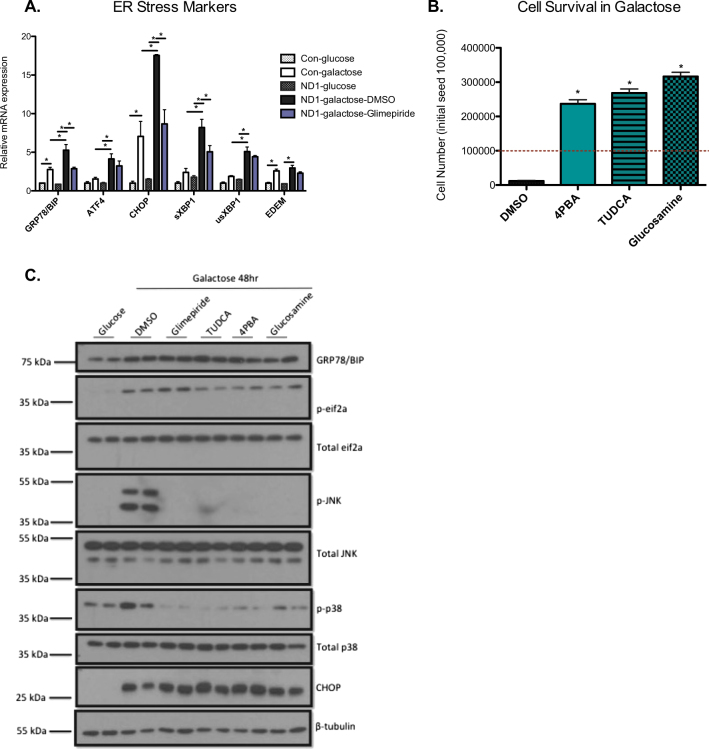
Galactose media initiates an ER stress response leading to p38 activation. **a** mRNA levels of ER stress markers for control or ND1 cells cultured in either glucose or galactose for 48 h, with or without glimepiride treatment. Results shown as mean ± SEM (*n* = 3); *p < 0.05. **b** Cell survival of ND1 cells treated with DMSO or an ER stress inhibitor (4PBA, TUDCA, or glucosamine) after 72 h in galactose. Red dashed line indicates initial seeding density. Results shown as mean ± SEM (*n* = 3); *p < 0.05. **c** Immunoblot demonstrating GRP78 (BIP), CHOP, p-eif2a, p-JNK, and p-p38 protein levels of ND1 cells treated with either DMSO in glucose or galactose, or ND1 cells treated with glimepiride or an ER stress inhibitor (4PBA, TUDCA, or glucosamine) in galactose for 48 h

In addition to identifying an increase in inflammatory markers, global proteomics analysis also demonstrated that cells treated with glimepiride had reduced levels of HSP70 members, HERP1, ATF4, and CHAC1, all of which are proteins that are responsive to ER stress signaling (Fig. 2b). Moreover, ER stress is also known to induce apoptosis via stress kinases such as p38 and JNK^15^. To determine whether ER stress may play a role in ND1 cell death, we measured mRNA levels of several ER stress genes in both control and ND1 cells. ND1-mutants had increased levels of ER stress markers, such as *Bip/Grp78*, *Atf4*, *Chop*, and *XBP1* compared to control cells in galactose. Furthermore, treatment with glimepiride partially attenuated *Bip/GRP78*, *Chop*, and *sXBP1* mRNA levels (Fig. 3a). To determine if ER stress signaling is a stimulant in galactose-induced ND1 cell death, we treated ND1 cells with the chemical chaperones 4-PBA and TUDCA as well as glucosamine and measured cell viability and MAPK activation^11,22^. TUDCA, 4-PBA, and glucosamine all resulted in an increase in cell survival in galactose and prevented eif2a, JNK, and p38 activation, although they did not significantly alter BIP/GRP78 or CHOP protein levels (Fig. 3b, c). Taken together, this data indicates that blocking ER stress is sufficient to rescue ND1 cells from galactose-induced cell death and that p38 plays a key role in this process.

### Mitochondrial CI missense mutant cells are more vulnerable to ER stress-induced cell death, which can be rescued by glimepiride

**Fig. 4:**
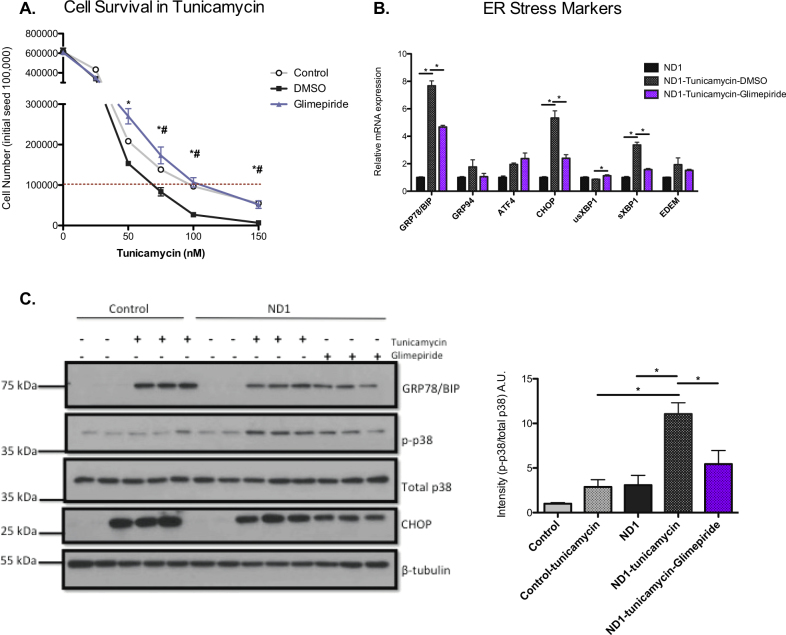
Glimepiride protects CI-deficient cells from ER stress-induced cell death. **a** Cell survival curve of control and ND1 cells treated with DMSO or glimepiride for 48 h in the presence of tunicamycin at different dosages. *ND1 glimepiride vs DMSO is significant. ^#^Control vs ND1 is significant. *^/#^*p* < 0.05. Results shown as mean ± SEM (*n* = 3). **b** mRNA levels of ER stress markers for ND1 cells in glucose conditions and either with tunicamycin or tunicamycin plus glimepiride. Results shown as mean ± SEM (*n* = 3); *p < 0.05. **c** Immunoblot of GRP78 (BIP), CHOP, and p-p38 levels in control and ND1 cells in glucose with or without tunicamycin along with ND1 cells treated with tunicamycin + glimepiride along with densitometry of p-p38/total p38 levels. Results shown as mean ± SEM (*n* = 3); *p < 0.05

To test whether ND1 cells are more sensitive to ER stress-induced cell death and whether glimepiride works by ameliorating ER stress, we treated control and ND1 cells with tunicamycin in glucose conditions with or without glimepiride. Tunicamycin treatment led to a dose-dependent induction of cell death in ND1-mutant cells, while glimepiride treatment increased cell survival at high tunicamycin concentrations (75–150 nM) and cell proliferation at lower concentrations (50 nM). Additionally, an increase in ND1 cell tunicamycin-sensitivity was observed when compared with control cells (Fig. 4a). We next measured ER stress markers in ND1-mutants treated with tunicamycin with either DMSO or glimepiride. Similarly to the effect of glimepiride treatment in galactose, several markers of ER stress including BIP, CHOP, and sXBP1 were significantly downregulated with glimepiride treatment (Fig. 4b). Furthermore, western blot analysis showed a reduction in GRP78/BIP, CHOP, and p-p38 with glimepiride treatment. As expected, p-p38 levels were also lower in control cells treated with tunicamycin than ND1 cells treated with tunicamycin (Fig. 4c). These results suggest that while galactose and tunicamycin elicit an ER stress response in both mutant and control cybrids, it is only an essential mediator of cell death in cells harboring the mitochondrial ND1 mutation.

**Fig. 5:**
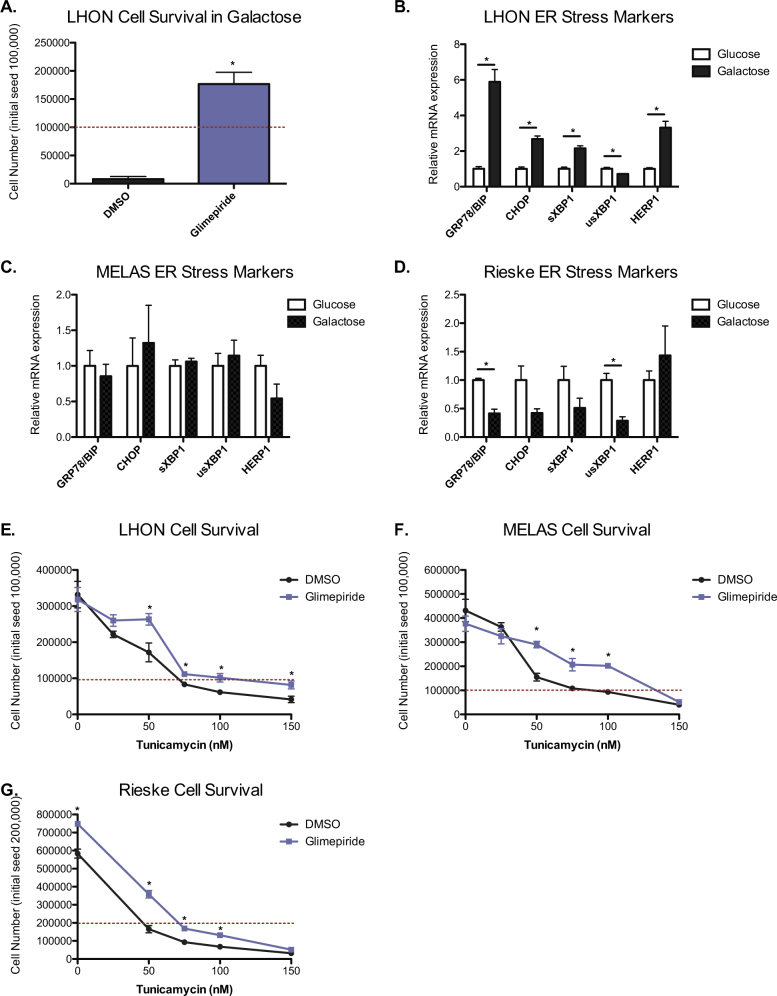
Glimepiride protects mitochondrial-mutant cells from ER stress- induced cell death. **a** Cell survival of LHON cells treated with DMSO or glimepiride in galactose for 72 h. Red dashed line indicates initial seeding density. Results shown as mean ± SEM (*n* = 3); *p < 0.05. **b** mRNA levels of ER stress markers for LHON cells cultured in either glucose or galactose for 48 h. Results shown as mean ± SEM (*n* = 3); *p < 0.05. **c** mRNA levels of ER stress markers for MELAS cells cultured in either glucose or galactose for 48 h. Results shown as mean ± SEM (*n* = 3). **d** mRNA levels of ER stress markers for Rieske KO cells cultured in either glucose or galactose for 48 h. Results shown as mean ± SEM (*n* = 3); *p < 0.05. **e** Cell survival curve of control and LHON cells, **f** MELAS, or **g** Rieske KO cells treated with DMSO or glimepiride for 48 h in the presence of tunicamycin at different dosages. Results shown as mean ± SEM (*n* = 3); *p < 0.05

We next tested a complex III Rieske knockout mouse fibroblast cell line and two additional human cybrid cell lines (MELAS and LHON) harboring a mitochondrial tRNA or mitochondrial CI ND6 mutation respectively, to determine if glimepiride could be used to rescue other mitochondrial mutant cells from galactose-induced cell death. Interestingly, we found that glimepiride was only able to rescue the other CI-deficient cells, LHON cells (Fig. 5a). Both the Rieske fibroblasts and MELAS cybrid cells underwent complete cell death within 12 h with or without glimepiride treatment (data not shown). In an attempt to explain these results, we measured ER stress markers in glucose and galactose conditions for all three cell lines. While LHON cells demonstrated a similar phenotype to ND1 cells when placed in galactose, neither Rieske nor MELAS cells exhibited an increase in ER stress markers in galactose (Fig. 5b, c, d). Since glimepiride conveys rescue in CI-deficient cells by inhibiting the ER stress response, it is not surprising that glimepiride failed to protect Rieske and MELAS cells and suggests that the mechanism of cell death is different in these cells. To further demonstrate however, that glimepiride can rescue cells from ER stress-induced cell death, we cultured all three cell lines in glucose media containing tunicamycin at different concentrations. Similarly to ND1 cells, glimepiride attenuated cell death at higher concentrations of tunicamycin and increased proliferation at lower concentrations (Fig. 5e, f, g). Taken together, these data shows that glimepiride effectively rescues mitochondrial CI-mutants from cell death during periods of energetic stress and more broadly, protects cells from ER stress-induced cell death.

### Galactose induces an ER Stress response leading to the activation of IRE1α and p38 in mitochondrial CI-mutant cells

**Fig. 6:**
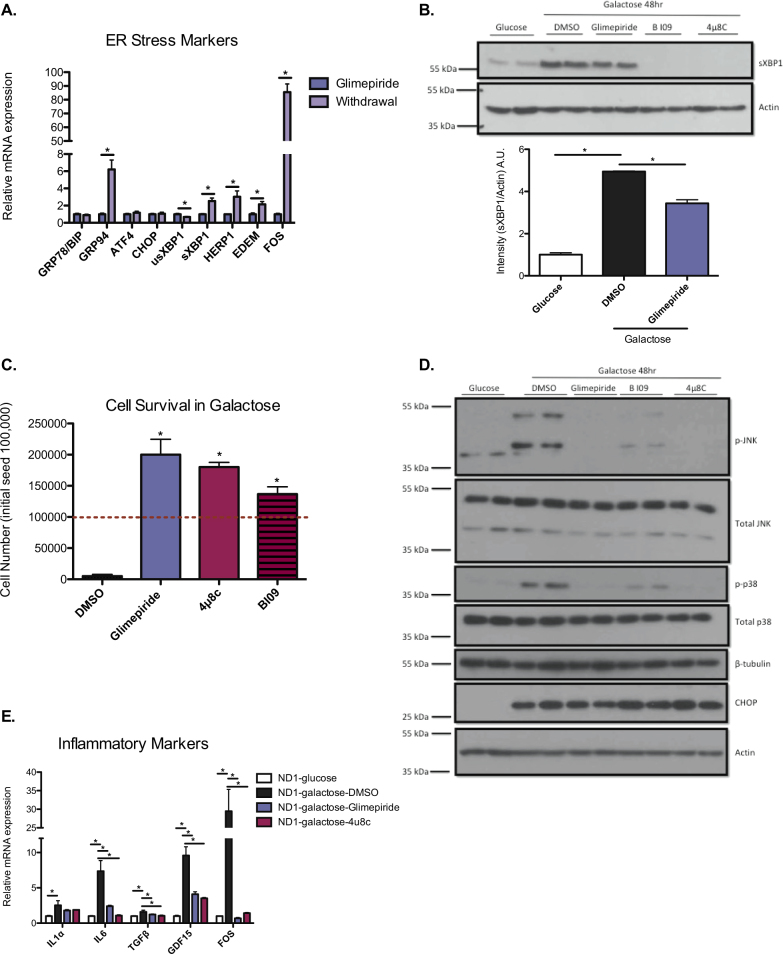
IRE1α couples ER stress to activation of p38 and JNK protein kinases. **a** mRNA levels of ER stress markers of ND1 cells either continuously cultured in glimepiride, or 48 h after glimepiride withdrawal. Results shown as mean ± SEM; *p < 0.05 (*n* = 3). **b** Spliced XBP1 levels in ND1 cells cultured in glucose, galactose, or galactose and treatment with glimepiride or IRE1α inhibitors. Results shown as mean ± SEM; *p < 0.05 (*n* = 2). **c** Cell survival of ND1 cells treated with DMSO, glimepiride, B I09, or 4 μ 8C in galactose. Red dashed line indicates initial seeding density. Results shown as mean ± SEM; *p < 0.05 (*n* = 3). **d** Western blot analysis of p-JNK, p-p38, and CHOP levels in cells cultured in galactose for 48 h with glimepiride or IRE1α inhibitors. **e** mRNA levels of inflammatory markers from ND1 cells cultured in glucose or galactose with DMSO, glimepiride or 4 μ8C analyzed by qPCR. Results shown as mean ± SEM; *p < 0.05 (*n* = 3)

Initially when cells are cultured in galactose media, a global induction of the UPR pathway is observed (Fig. 3a); therefore, in an effort to delineate which arm of the ER stress response is responsible for cell death we performed a withdrawal experiment. Since cells are dependent on glimepiride for cell survival (Fig. 1e), we cultured cells in galactose with glimepiride for 1 week and then either removed the drug or continued treatment for another 48 h and measured mRNA levels of ER stress genes. Interestingly, the only genes that were significantly increased by glimepiride withdrawal were *GRP94*, *HERP1*, *EDEM*, and *XBP1*, all of which are induced by the IRE1α branch of the ER stress pathway (Fig. 6a)^23^. Notably, in response to ER stress IRE1α is a central mediator that activates the inflammatory kinases JNK and p38, suggesting that the IRE1α branch of the UPR might cause mitochondrial energetic-dependent cell death in ND1-mutant cells. To test the vulnerability of these cells to IRE1α activation, we treated ND1 cells with the IRE1α specific inhibitors, 4μ8C and B I09, in galactose for 72 h and measured cell viability. Strikingly, both IRE1α inhibitors completely blocked splicing of XBP1 (a target of IRE1α) in galactose, and led to a significant increase in cell survival compared to cells treated with DMSO (Fig. 6b, c). Interestingly, while IRE1α inhibitors suppress both XBP1 splicing and stress kinases, sulfonylureas completely block the inflammatory pathway while only mildly repressing XBP1 splicing in comparison to the IRE1α inhibitors (Fig. 6b, d). We next measured whether IRE1 inhibition can blunt the increase in inflammatory markers driven by galactose to a similar degree as glimepiride treatment and found that treating ND1 cells with 4μ8C led to a significant decrease in *IL6*, *GDF-15*, and *FOS* and a mild decrease in *IL1*α mRNA levels, comparable to that of glimepiride (Fig. 6e). These results indicate that during energetic stress, mitochondrial CI-mutant cells activate the ER stress IRE1α pro-inflammatory pathway causing cell death.

## Discussion

It has been described that not only do the ER and mitochondria physically interact, but that dysfunction of one organelle can contribute to the dysfunction of the other^24,25^. Here we demonstrated that in addition to mitochondrial mutations leaving cells vulnerable to energetic stress-induced cell death; they also render the cell unable to cope with elevated ER stress. We show a shift from glucose to galactose elicits an ER stress response in both control and mitochondrial CI-deficient cells; however, while control cells are able to resolve this stress, CI-mutants have an increased sensitivity to ER stress leading to activation of p38 by IRE1α and eventually cell death. One possibility for these differences in cell fate, is that an increase in ATP production is necessary for the ATP-consuming process of chaperone protein folding and while control cells are able to increase ATP levels to meet this demand, mitochondrial mutants are not.

Previously, we have shown that one approach to rescuing CI-deficient cells involves metabolic reprogramming through BRD4 inhibition, which allows for remodeling of the mitochondrial proteome and increases the utilization of FADH_2_, allowing substrates to bypass a mutant CI^9^. Here, we have identified an alternative approach to rescuing CI-deficient cells, independent of increasing ETC activity and ATP synthesis. We found that treating CI-mutants with glimepiride was sufficient to rescue cells cultured in either galactose or glucose media containing tunicamycin. Glimepiride conveyed rescue by suppressing ER stress levels and preventing prolonged activation of IRE1α. We demonstrate that while glimepiride has some effect on XBP1 splicing, it predominately acts by inhibiting the kinase activity of IRE1α to activate MAPK p38 and JNK. Of note, while glimepiride was able to rescue cells with a CI-missense mutation, it was unable to rescue cells containing a CIII or tRNA mutation. This may be attributed to the severity of the mutations, as MELAS and Rieske cells do not maintain any detectable respiration as opposed to ND1 and ND6 mutants (data not shown).

Of interest, a heavily debated topic for decades has been the existence of mitochondrial K+ (ATP) channels and whether they play a role in mitochondrial-induced apoptosis^26^. Recent findings suggest that the mitochondrial K+ (ATP) channel may be composed of multiple mitochondrial proteins including some components of the permeability transition pore; however, the molecular structure and protein interactions that compose the channel remain unknown^27^. Although we have not investigated the effect of glimepiride on the mitochondrial K+ (ATP) channel, other studies have demonstrated that glimepiride has no effect on mitochondrial K+ (ATP) channels in ventricular myocytes using similar concentrations, and that K+ (ATP) channel openers, not inhibitors, are proposed to be antiapoptotic^27,28^.

While the primary role of the UPR is to decrease ER stress and restore homeostasis, the UPR also plays an important role in regulating inflammation. This has been described in various pathological conditions such as metabolic diseases, neurodegenerative disorders, and atherosclerosis^29^. The UPR triggers its inflammatory response through NF-κB, JNK, and inflammasome activation as well as through transcriptional control of cytokine genes^30–32^. Here, we have demonstrated that several cytokines are significantly increased upon culturing CI-deficient cells in galactose conditions. Furthermore, treating cells with either glimepiride or a p38 inhibitor blunts this increase, demonstrating that p38 is responsible for the inflammatory response. While it is unclear, how the inflammatory response further aggravates and contributes to the mitochondrial dysfunction and cell death, it has been reported that certain cytokines negatively impact OXPHOS^33,34^. Likewise, mitochondrial dysfunction has also been shown to increase inflammation, although this has been primarily attributed to increases in ROS^35^. Interestingly, GDF-15 has been described as a biomarker for mitochondrial dysfunction. Several studies suggest that GDF-15 induction promotes metabolic adaptation and can be driven by p38 kinase activation of CHOP^36^. Both global proteomics and qPCR analysis suggest that GDF-15 is similarly regulated by p38 kinase, as ND1-mutants show elevated levels of GDF-15 when cultured in galactose, and glimepiride and p38 inhibition suppress this elevation. These results raise the question of whether ER stress is an extenuating factor in disease progression in patients with mitochondrial disorders and whether addressing any underlying ER stress could be beneficial.

Overall, we performed an unbiased high-throughput chemical screen in which sulfonylureas were identified as compounds that can rescue mitochondrial CI-deficient cells from energetic stress conditions. Although it is unlikely that sulfonylureas will be used as a therapeutic option for treating patients with mitochondrial diseases due to their control over insulin secretion, this study reveals that IRE1α and p38 inhibitors may serve as more practical compounds in the future treatment of these disorders. In addition, we further highlight the importance of intracellular-crosstalk between the ER and the mitochondria, particularly during alterations in nutrient availability. Importantly, this study reveals that there may be additional strategies worth exploring that may rescue mitochondrial deficiencies besides strictly increasing ATP production.

## Materials and methods

### Cell culture and treatments

Unless otherwise stated, human ND1-mutant (3796A>G) cybrid, human MELAS (A3243G-Leucine tRNA) cybrid, human LHON ND6 (G14459A) cybrids, and control cybrid cells were routinely cultured in Dulbecco’s modified Eagle’s medium (DMEM) (Gibco) containing 10% fetal bovine serum, 100 U/mL penicillin and streptomycin, 1 mM sodium pyruvate, and 4 mM l-glutamine. Mouse Rieske knockout cells were cultured in the same media, but also supplemented with 5 μg/mL uridine. Depending on the experiment, media also contained either 10 mM galactose or 25 mM glucose. For cell survival experiments: 100,000 cells were seeded directly in galactose-media with or without compounds or in glucose media containing varying dosages of tunicamycin. Cells were trypsinized and counted 48 or 72 h later unless otherwise stated.

Drug treatments included: 25 μM glimepiride unless otherwise stated (Santa Cruz Biotechnology Inc.), 25 μm glyburide (Sigma-Aldrich), 25 μM repaglinide (Sigma-Aldrich), 25 μM SB203580 (Santa Cruz Biotechnology Inc.), 2 μM SB202190 (Sigma-Aldrich), 20 μM SP600125 (Sigma-Aldrich), 1 mM sodium 4-phenylbutyrate, 500 μM Tauroursodeoxycholic acid sodium salt (Fisher Scientific), 100 μM d-(+)-glucosamine (Sigma-Aldrich), 25 μM B I09 (Tocris), 20 μM 4 μ8C (7-hydroxy-4-methyl-2-oxo-2*H*-chromene-8-carbaldehyde, Sigma), tunicamycin (Sigma-Aldrich) at varying dosages. Drug treatments were typically performed for either 48 or 72 h in galactose media unless otherwise noted.

CRISPR constructs: The lentiCRISPR v2 plasmid (addgene #52961) was used to clone all CRISPR constructs. Guide sequences for Kir6.2 were as follows. Kir6.2#1, 5′-CACCGAAGTGACTATTGGCTTTGGG-3′; Kir6.2#2, 5′-CACCGGAGTGGATGCTGGTGACAC-3′.

### High-throughput chemical screen

A total of 1500 human ND1-mutant (3796A>G) cybrid cells were seeded per well of a 384-well plate (Corning #3570) in either 40 μL volume of DMEM media (Gibco) without glucose, with 10 mM galactose and 2% FBS or DMEM media (Gibco) with 1.25 mM Glucose, 10 mM galactose, and 2% FBS using a Matrix well-mate. Both negative and positive control wells also contained 0.3% DMSO while experimental wells had 100 nL of compound added via pin transfer immediately after seeding. Concentrations of compounds varied; however, most compounds were administered to give a final concentration of 10 or 25 μM. Cells were then incubated for 72 h at 37 °C with 5% CO_2_. Following the 72-hour incubation period, the 384-well plates were briefly centrifuged for 1 min at 1000 rpm. The media was aspirated using an aspiration wand and fresh media (DMEM only), was added to the cells (40 μL/ well) using the Matrix well-mate. Fifteen-microliters of Cell Titer Glow (Promega) substrate was then added to each well using the Matrix well-mate. The plates were gently vortexed for 10 s and then centrifuged for 1 min at 1000 rpm. The plates were then immediately read on the Perkin Elmer EnVision plate reader and the luminescence was detected for each individual well. Two replicates were prepared as described at the same time per library plate. A *Z′* score was determined for each experimental well. This was done using the following formula: *Z′*  = (*x* – *μ*)/*σ*; where *x* is the luminescence value of the well to be standardized, *μ* is the mean of the experimental well population of plate, and *σ* is the standard deviation of the experimental well population of plate. Each replicate was calculated separately. Potential hits were determined to have *Z′* score of 1.8 or higher for both replicates. A score of 1.8–3 was considered a weak hit, >3–4 was considered a medium hit, and >4 was considered a strong hit.

### Western blot analysis

Cells were harvested and lysed in radioimmunoprecipitation assay (RIPA) buffer containing a cocktail of protease (Roche) and phosphatase inhibitors (Roche), separated by SDS-PAGE, and transferred to Immobilon-P membranes (Millipore). The following antibodies were used: tubulin (Millipore), cleaved caspase 3 (Asp 175) (Cell Signaling), cleaved PARP (Asp 214) (Cell Signaling), total PARP (Cell Signaling), total JNK (Cell Signaling), phospho-JNK (T183/Y185) (Cell Signaling), total p38 (Cell Signaling), phospho-p38 (T180/Y182) (Cell Signaling), total MKK4 (Cell Signaling), phospho-MKK4 (Ser257/Thr261) (Cell Signaling), GRP78 (BD Biosciences), total eif2a (Cell Signaling), and phospho-eif2a (Cell Signaling), XBP-1 s (Cell Signaling), CHOP (Cell Signaling), and Kir6.2 (Santa Cruz). Quantification of western blot analysis was done using ImageJ.

### Gene expression

Total RNA from cultured cells was purified using TRIzol (Invitrogen) for cDNA synthesis (Applied Biosystems high-capacity kit). Relative mRNA expression was measured by quantitative PCR (qPCR) using SYBR green dye (Applied Biosystems) and specific primers for genes of interest. All primers and sequences are available upon request.

### ADP/ATP ratio

ADP/ATP ratio was measured according to manufacture’s instructions using a bioluminescence assay kit (BioVision ApoSensor).

### ROS generation

ROS-sensitive probe CM-H_2_DCFDA (Molecular Probes) was diluted in cell culture media and added to cells after 2 days of appropriate drug treatment. Cells were incubated at 37 °C for 30 min and washed twice with PBS. Cells were then trypsinized and pelleted, then resuspended in PBS. Fluorescence was examined by flow cytometry on a BD FACS CANTOII flow cytometer (BD Bioscience) using a 488-nm argon excitation laser.

### Mitochondrial membrane potential

Tetramethylrhodamine, methyl ester (TMRM) dye (Thermo Fisher Scientific) was diluted in cell culture media and added to cells after 2 days of indicated culture conditions. Cells were incubated at 37 °C for 30 min and washed twice with PBS. Cells were then trypsinized and pelleted, then resuspended in PBS. Fluorescence was examined by flow cytometry on a BD FACS CANTOII flow cytometer (BD Bioscience) using a 488-nm argon excitation laser.

### Oxygen consumption measurements

ND1-mutant cells were cultured in galactose either in DMSO or 25 μM glimepiride for 48 h. Cells were collected and mitochondria was isolated in a sucrose/mannitol buffer containing 0.5% (w/v) fatty acid free BSA (pH 7.2). Mitochondria were spun down and resuspended in a sucrose/mannitol based mitochondrial assay solution (MAS). A total of 50 μL of 40 μg of mitochondria were seeded per well in a seahorse plate on ice. Mitochondria were spun down at 2000 × *g* for 20 min. Afterwards, 450 μL of MAS buffer containing 2 mM malate, 10 mM pyruvate, and 0.5 mM ADP was added to each well. The seahorse plate containing mitochondria were briefly place at 37 °C and then transferred to the XF24 Analyzer.

### Mitochondrial enzymatic activities

Crude mitochondrial fractions were isolated from ND1-mutant cells 48 h after treatment with glimepiride by differential centrifugation. Activities were determined for complexes I, II, IV, and CS using a SpectraMax M5 spectrophotometer by Molecular Devices. Activity levels were normalized to citrate synthase activity. All enzymatic activity assays were performed as described by Spinazzi et al.^37^.

### Statistical analysis

All statistics are described in figure legends. In general, for two comparisons, an unpaired Student’s *t*-test was used. For multiple comparisons, one-way ANOVA with Bonferroni post-test was applied. Three replicates per treatment were used as a sample size unless otherwise stated in the figure legend. All western blot analyses were repeated minimally three times. Statistical significance is represented with an asterisk where *P* < 0.05.

### Proteomics protein extraction and protease digestion

Cell pellets were combined 1:1 with SDS lysis buffer (4.0 % SDS w/v, 250 mM NaCl, PhosStop (Roche) phosphatase inhibitors, EDTA-free protease inhibitor cocktail (Promega) and 50 mM HEPES, pH 8.5). Extracts were reduced with 5 mM DTT (57 °C for 30 min) and cysteine residues alkylated with iodoacetamide (14 mM) in the dark (45 min). Extracts were purified by methanol–chloroform precipitation and subsequent ice cold acetone washes. Pellets were resuspended in 8 M urea containing 50 mM HEPES, pH 8.5, and protein concentrations were measured by BCA assay (Thermo Scientific) prior to protease digestion. A total of 200 µg of protein extracts were diluted to 4 M urea and digested with LysC (Wako) in a 1/200 enzyme/protein ratio overnight. Digests were diluted further to a 1.5 M urea concentration and trypsin (Promega,) was added to a final 1/250 enzyme/protein ratio for 6 h at 37 °C. Digests were acidified with 20 µL of 20% formic acid (FA) to a pH ~2 and subjected to C18 solid-phase extraction (SPE) (50 mg, Sep-Pak, Waters).

### Isobaric tag labeling

Isobaric labeling of peptides was performed using a 6-plex tandem mass tag (TMT) reagents (Thermo Fisher Scientific). TMT reagents (5 mg) were dissolved in 250 µL dry acetonitrile (ACN) and 10 µL was added to 100 µg of peptides dissolved in 100 µL of 200 mM HEPES, pH 8.5. After 1 h (RT), the reaction was quenched by adding 8 µL of 5% hydroxylamine. Labeled peptides were combined, acidified with 20 µL of 20 % FA (pH ~2) and concentrated via C_18_ SPE on Sep-Pak cartridges (50 mg).

### Basic pH reversed-phase HPLC

TMT labeled peptides were solubilized in buffer A (5% ACN, 10 mM ammonium bicarbonate, pH 8.0) and separated by an Agilent 300 Extend C18 column (5 μm particles, 4.6 mm ID, and 220 mm in length). Using an Agilent 1100 binary pump coupled with a degasser and a photodiode array (PDA) detector (Thermo Scientific), a 45 min linear gradient from 18 to 40% acetonitrile in 10 mM ammonium bicarbonate pH 8 (flow rate of 0.8 mL/min) separated the peptide mixtures into a total of 96 fractions (37 s). Ninety-six fractions were consolidated into 12 samples in a checkerboard manner, acidified with 10 µL of 20% formic acid and vacuum dried. Each sample was re-dissolved in 5% FA, desalted via StageTips, dried via vacuum centrifugation, and reconstituted for LC-MS/MS analysis.

### Oribtrap fusion parameters

All MS analysis was performed on an Oribtrap Fusion Lumos (Thermo Fischer Scientific) coupled to a Proxeon nLC-1200 ultra-high pressure liquid chromatography (UPLC) pump (Thermo Fisher Scientific). Peptides were separated onto a packed 100 µM inner diameter column containing 0.5 cm of Magic C4 resin (5 μm, 100 Å, Michrom Bioresources) followed by 40 cm of Sepax Technologies GP-C_18_ resin (1.8 μm, 120 Å) and a gradient consisting of 6–30% (ACN, 0.125% FA) over 125 min at ~450 nL/min. The instrument was operated in data-dependent mode with a 60 s (±7 ppm window) expiration time, with FTMS^1^ spectra collected at 120,000 resolution with an AGC target of 500,000 and a max injection time of 100 ms. The ten most intense ions were selected for MS/MS and precursors were filtered according to charge state (required > 1 z). Monoisotopic precursor selection was enabled. Isolation width was set at 0.7 *m/z*. ITMS^2^ spectra were collected at an AGC of 18,000, max injection time of 120 ms and CID collision energy of 35%. For the FTMS^3^ acquisition, the Orbitrap was operated at 30,000 resolution with an AGC target of 50,000 and a max injection time of 250 ms and an HCD collision energy of 55%. Synchronous-precursor-selection (SPS) was enabled to include 10 MS^2^ fragment ions in the FTMS^3^ spectrum.

### Data processing and MS^2^ spectra assignment

A compendium of in-house software was used to convert.raw files to mzXML format, as well as to correct monoisotopic *m/z* measurements and erroneous charge state assignments. Assignment of MS/MS spectra was performed using the Sequest algorithm. A protein sequence database containing Human Uniprot database (downloaded 11/2015) as well as known contaminants such as human keratins and reverse protein sequences were appended. Sequest searches were performed using a 20 ppm precursor ion tolerance, requiring trypsin protease specificity, while allowing up to two missed cleavages. TMT tags on peptide N termini/lysine residues (+229.162932 Da) and carbamidomethylation of cysteine residues (+57.02146 Da) were set as static modifications while methionine oxidation (+15.99492 Da) was set as variable modifications. An MS^2^ spectra assignment false discovery rate (FDR) of <1% was achieved by applying the target-decoy database search strategy and filtered using an in-house linear discrimination analysis algorithm with the following peptide ion and MS^2^ spectra metrics: XCorr, peptide ion mass accuracy, charge state, peptide length, and missed-cleavages. Peptides were further filtered a 1% protein-level false discovery rate for the final dataset.

### Calculation of TMT reporter ion intensities

For quantification, a 0.03 *m/z* (6-plex TMT) window centered on the theoretical *m/z* value of each reporter ion, with the maximum signal intensity from the theoretical *m/z* value was recorded. Reporter ion intensities were adjusted based on the overlap of isotopic envelopes of all reporter ions as per manufacturer specifications. Total signal to noise values for all peptides were summed for each TMT channel (300 minimum) and all values were normalized to account for variance in sample handling.

## Electronic supplementary material

Supplementary Figure 1

Supplementary Figure 2

Supplementary Figure 3

Supplementary Figure 4

Supplementary figure legends
