# Supplementary material for: Inhibition of the ER stress IRE1α inflammatory pathway protects against cell death in mitochondrial complex I mutant cells
Source: Cell Death Dis. 2018 May 31;9(6):658. doi: 10.1038/s41419-018-0696-5 (PMC5981317; doi:10.1038/s41419-018-0696-5)
Supplement: Supplementary file 5 — Supplementary figure legends [file 41419_2018_696_MOESM5_ESM.docx]

**Supplementary Figure Legends**

**Supplementary 1. Sulfonylurea treatment slows long-term cell growth in control cells**

Control cell growth over two-weeks with DMSO or glimepiride treatment. Results shown as mean ±SEM (n=2); *p<0.05.

**Supplementary 2. Sulfonylureas’ rescue is independent of the K+(ATP) channel**

1. Dose response survival curve of ND1 cells treated with glimepiride. Red dashed line indicates initial cell seed. Results shown as mean ±SEM (n=3).
2. Western blot analysis of Kir6.2 protein levels of V2 control and Kir6.2 CRISPR ablation in ND1 cells. Results shown as mean ±SEM; *p<0.05 (n=2).
3. Cell survival of V2 control and Kir6.2 depleted cells in galactose. NS= not significant. Results shown as mean ±SEM (n=3).

**Supplementary 3. MAPK activation and inflammatory gene induction is specific to ND1-deficient cells**

1. Western blot analysis of p-JNK, p-p38, total JNK, and total p38 levels in control cells cultured in glucose or galactose compared to ND1-mutants grown in glucose or galactose for 48 hours.
2. qPCR analysis demonstrating mRNA levels of different inflammatory cytokines in control cells or ND1 cybrid cells cultured in either glucose or galactose for 48 hours. Results shown as mean ±SEM (n=3); *p<0.05.

**Supplementary 4. Sulfonylureas do not alter mitochondrial bioenergetics**

1. ATP/ADP levels in ND1 cells cultured in galactose for 8, 16, 24, and 48 hours with either DMSO or glimepiride. Results shown as mean ±SEM (n=3).
2. Mitochondrial enzymatic activity of complexes I, II and IV of ND1 cells cultured in galactose with either DMSO or glimepiride. Complex activity normalized to citrate synthase levels. Results shown as mean ±SEM; *p<0.05 (n=3).
3. Citrate synthase levels used for normalization in B. Results shown as mean ±SEM (n=3).
4. Basal oxygen consumption levels of cells treated with DMSO or glimepiride in galactose. Results shown as mean ±SEM (n=3).
5. DCF fluorescence intensity (ROS levels) in ND1 cells cultured in glucose or galactose with DMSO or glimepiride. Results shown as mean ±SEM; *p<0.05 (n=3).
6. Mitochondrial membrane potential measured in ND1 cells cultured in glucose or galactose with DMSO or glimepiride. Results shown as mean ±SEM; *p<0.05 (n=3).
